# Anticoagulation in chronic kidney disease: from guidelines to clinical practice

**DOI:** 10.1002/clc.23196

**Published:** 2019-05-28

**Authors:** Viviana Aursulesei, Irina Iuliana Costache

**Affiliations:** ^1^ 1st Medical Department, Division of Cardiology, Faculty of Medicine Grigore T. Popa University of Medicine and Pharmacy Iasi Romania

**Keywords:** chronic kidney disease, direct oral anticoagulants, heparin, warfarin

## Abstract

**Background:**

Chronic kidney disease (CKD) is a major global public health problem, being closely connected to cardiovascular disease. CKD involves an elevated thromboembolic risk and requires anticoagulation, but the high rates of hemorrhage render it quite challenging.

**Hypothesis:**

There are no consensus recommendations regarding anticoagulation in CKD. Due to the currently limited data, clinicians need practical clues for monitoring and optimizing the treatment.

**Methods:**

Based on the available data, this review outlines the benefit‐risk ratio of all types of anticoagulants in each stage of CKD and provides practical recommendations for accurate dosage adjustment, reversal of antithrombotic effect, and monitoring of renal function on a regular basis.

**Results:**

Evidence from randomized controlled trials supports the efficient and safe use of warfarin and direct oral anticoagulants (DOACs) in mild and moderate CKD. On the contrary, the data are poor and controversial for advanced stages. DOACs are preferred in CKD stages 1 to 3. In patients with stage 4 CKD, the choice of warfarin vs DOACs will take into consideration the pharmacokinetics of the drugs and patient characteristics. Warfarin remains the first‐line treatment in end‐stage renal disease, although in this case the decision to use or not to use anticoagulation is strictly individualized. Anticoagulation with heparins is safe in nondialysis‐dependent CKD, but remains a challenge in the hemodialysis patients.

**Conclusions:**

Although there is a need for cardiorenal consensus regarding anticoagulation in CKD, adequate selection of the anticoagulant type and careful monitoring are some extremely useful indications for overcoming management challenges.

## 1 INTRODUCTION

Chronic kidney disease (CKD) is a major global public health problem, being closely connected to cardiovascular disease (CVD). Arterial hypertension and diabetes mellitus are the primary causes of CKD, while CKD is recognized as an independent risk factor for the onset of CVD. The latest United States Renal Data System report states that the prevalence of any CVD is double in patients with CKD, estimated at 69.8% vs 34.8% in the general population.^1^ Also, if microalbuminuria is detected and glomerular filtration rate (eGFR) is less than <60 mL/min/1.73m^2^, there is an increased risk of cardiovascular events and mortality. The relationship is early and gradual across the entire spectrum of CKD (Table 1), causing the majority of patients to succumb to cardiovascular complications.^2^

**Table 1:** Stages of chronic kidney disease based on glomerular filtration rate (eGFR) categories^2^

Stage	1	2	3a	3b	4	5
eGFR category	Normal and high	Mild reduction	Mild‐moderate reduction	Moderate‐severe reduction	Severe reduction	Kidney failure
eGFR (mL/min/1.73m^2^)	≥90	60‐89	45‐59	30‐44	15‐29	<15

The risk of atrial fibrillation (AF) and acute coronary syndrome (ACS) is double in patients with eGFR <60 mL/min/1.73m^2^.^3^ The study model most relevant to clinical practice is AF in CKD. The prevalence of AF increases with the decline in renal function, the prevalence of end‐stage renal disease (ESRD) being two or even three times higher than in the general population.^4^ Depending on the studied cohorts, the prevalence of AF in CKD patients was estimated to range between 12% and 18% compared to 7%‐8% in the general population over 65 years of age. The prevalence of AF remains high (11.6%) in dialysis‐dependent CKD, and 12 months after kidney transplantation, the risk of AF occurrence increases up to 35.6% per 1000 patient years.^5^ On the other hand, large prospective cohort studies demonstrate the relationship between incident AF and an 80% higher risk of decline in eGFR and a 116% higher risk of proteinuria detection.^6^ In the chronic renal insufficiency cohort prospective study, AF led to a 3‐fold higher risk of progression to ESRD.^7^ The association between AF and CKD engenders a much higher thromboembolic risk, which in case of ischemic stroke ranges from 26% to 49%, depending on the study.^5^ Also, the relative risk of mortality increases by up to 66%.^6^ The association between ACS and CKD is also well documented in registries. Thus, 40% of non‐ST segment‐elevation myocardial infarction (NSTEMI) cases and 30% of ST segment‐elevation myocardial infarction (STEMI) cases associate eGFR <60 mL/min/1.73 m^2^, and mortality is double compared with general population.^3^ The risk of pulmonary venous thromboembolism (VTE) in CKD increases by 25%‐30% is constant in all CKD stages, and typically characterizes the nephrotic syndrome.^8^

## 2 WHAT IS THE UNDERLYING CONNECTION BETWEEN CKD AND CVD?

The bidirectional relationship between CVD and CKD is due to the intervention of major cardiovascular risk factors shared by both disorders. Concurrently, CKD entails factors that are frequently involved in uremia, such as systemic inflammation, oxidative stress, activation of the renin‐angiotensin‐aldosterone system, malnourishment, anemia, microalbuminuria, hyperhomocysteinemia, as well as hyperparathyroidism, abnormalities in bone and mineral metabolism (in which phosphorus, fibroblastic/fibroblast growth factor 23, vitamin D deficiency play an important role), the particular profile of apolipoprotein isoforms, platelet hyperreactivity, all with impact on the cardiovascular system.^4^, ^9^, ^10^ This results in a hypercoagulable state that generates arterial and venous thromboembolic complications, as well as the progression of kideny disease. The pathophysiological substrate involves the three components of Virchow's triad—stasis and turbulent blood flow, vascular endothelial injury, and hypercoagulability. Endothelial injury/dysfunction is an essential promoter of the proinflammatory, procoagulant and pro‐proliferative state.^9^, ^10^ In case of uremia, high levels of fibrinogen, thrombin‐antithrombin complexes, thrombomodulin, von Willebrand factor, plasminogen activator inhibitor 1 (PAI‐1), factor VII are markers of endothelial dysfunction. A particular mechanism in uremia is the intervention of homocysteine via thrombin activation, fibrin formation, and reduced release of tissue plasminogen activator from the endothelium. Via these mechanisms, along with PAI‐1, homocysteine causes a decreased fibrinolytic activity.^9^ Also, the platelets of uremic patients are dysfunctional due to the activation of certain microRNA‐altering mechanisms. This results in microparticles expressing tissue factor, the key‐element for the initiation of the coagulation cascade. PAI‐1 secretion links endothelial dysfunction to structural cardiovascular (CV) changes, as it promotes tissue fibrosis. Arterial stiffness occurs along with early onset atherosclerosis and vascular calcifications. The heart shows left ventricular hypertrophy and marked myocardial fibrosis with impact on coronary circulation, atrial and ventricular remodeling, and blood flow implicitly.^4^ The clinical consequence is an increased thromboembolic risk.

The paradox in CKD is the association between the high thromboembolic risk and major hemorrhagic risk with declining kidney function. Platelet hyperreactivity in the early stages is replaced by decreased platelet activity and impaired platelet‐vessel wall interaction. This is caused by the alteration of platelet‐dependent mechanisms involved in physiological hemostasis.^9^, ^10^ Overall, platelet adhesion and aggregation are reduced.^11^ The prohemorrhagic state is potentiated by CKD‐related anemia, extrinsic iatrogenic factors (nonsteroidal anti‐inflammatory drugs, antithrombotic medication, antibiotics, invasive procedures, dialysis methods), and gastrointestinal lesions.^4^ Epidemiologic studies confirm the elevated hemorrhagic risk, which can be 4.1 times higher in ischemic stroke and 10.7 times higher in intracerebral hemorrhage in dialysis patients.^12^

In CKD, the fragile balance between the risk of thromboembolic events and hemorrhage puts the population requiring anticoagulation treatment in a very difficult position for the following reasons:

- the need for anticoagulants is much higher in CKD;
- the population with advanced CKD stages is frequently excluded from controlled randomized trials, so there is no consistent evidence for the efficacy and safety of anticoagulants;
- there are no thromboembolic and hemorrhagic risk scores that adequately define individual risk;
- the risk‐benefit ratio is influenced by numerous variables specific to this subgroup;
- there are pharmacokinetic and pharmacodynamic features related to the impaired renal functions, and interaction with other drugs requiring adjustment of therapeutic regimens;
- the methods used to assess renal dysfunction vary between the studies, which obviously leads to contradictory results;
- there is no consensus on the recommendations for oral anticoagulation, and the use of a particular type of anticoagulant, especially in stages 4 to 5 CKD cannot be supported.

## 3 PROBLEMS AND SOLUTIONS

### 3.1 Pharmacokinetics and pharmacodynamics

#### 3.1.1 The oral anticoagulants

Oral anticoagulants (OACs) used in CKD do not differ from those used in general practice and are represented by vitamin K antagonists (VKAs) and direct OACs. However, their pharmacokinetic and pharmacodynamic features require the adjustment of therapeutic regimens (Table 2).^13^

**Table 2:** Pharmacokinetic properties of oral anticoagulants (adapted from Jain et al^13^ and Lutz et al^14^)

Oral anticoagulant	Mechanism of action	Prodrug	Pharmacokinetic properties		
			Metabolism	Dialyzable	Dose adjustment
Warfarin	Vitamin K antagonist	No	Predominantly via cytochrome P450 type 2C9 (CYP2C9)	No	No
Dabigatran	Direct inhibitor of free thrombin and fibrin‐bound thrombin	Yes	Renal excretion 80%	Yes	Yes
Rivaroxaban	Free and clot‐bound Xa factor inhibitor, prothrombinase activity inhibitor	No	Renal excretion 66%, 36% as unchanged drug	No	Yes
Apixaban	Free and clot‐bound Xa factor inhibitor	No	Metabolized in liver via CYP3A4, renal excretion 27% and in feces	Partial	No
Edoxaban	Free Xa factor and tissue factor inhibitor	No	10% hydrolyzed by carboxylesterase 1, 50% unchanged upon renal excretion	No	Yes

*VKAs* are still the most widely used, with the mention that is the warfarin for which the majority of clinical evidence was obtained. AF guidelines recommend warfarin differently in CKD. The American Guidelines American Heart Association/American College of Cardiology/Heart Rhythm Society (AHA/ACC/HRS) recommend warfarin in all CKD stages (stages 2‐3—Class 1, Level of Evidence (LOE) A, stage 4—Class IIb, LOE C, stage 5—Class IIa, LOE B). The Canadian Guidelines prefer warfarin in stage 4, and the European Society of Cardiology (ESC) guidelines do not provide specific information for a particular type of OAC in any CKD stage.^15^ Although the guidelines for AF or VTE do not recommend drug dose adjustment in CKD (Table 3), clinical studies reveal an increased hemorrhagic risk, particularly high within the first 30 to 90 days after initiation of treatment.^9^ Most major bleeding events are gastrointestinal, due to the high frequency of digestive lesions favored by uremia. The narrow therapeutic window and high interindividual variability often lead to supratherapeutic International Normalized Ratio (INR) in CKD. Moreover, response to warfarin is influenced by dietary rules, volemic variations, changes in drug metabolism, and drug‐drug interactions, vitamin K deficiency, treatment compliance.^13^ For this reason, to prevent the risk of hemorrhage requires an average reduction of warfarin doses by 10% in patients with eGFR between 30 and 59 mL/min/1.73m^2^ and by 19% in those with eGFR < 30 mL/min/1.73m^2^, in order to maintain INR ≤ 4.^11^ A particular aspect is the risk of acute renal failure at an INR threshold of >3, an entity known as warfarin‐induced nephropathy. It is defined as an unexplained increase in serum creatinine ≥0.3 mg/dL within 7 days of INR >3.0 in a patient treated with warfarin. The substrate is the glomerular hemorrhage via thrombin depletion and tubular obstruction with hematic cylinders. It is more frequent in CKD, with a mortality rate of up to 31% at 1 year.^9^, ^16^ Identifying at‐risk patients requires testing of CYP2C9 and VKORC1 polymorphisms in order to reduce the risk of overdose in case of warfarin sensitivity, method not introduced into current clinical practice.^17^ Dose adjustment is also necessary because in the liver plasma half‐life (*t*_1/2_) is shortened, the renal clearance is enhanced, and the interaction with other drugs is changed. Warfarin administration is even more difficult in dialysis patients, being associated with an increased risk of thromboembolic events and hemorrhage.^13^, ^16^ Ultimately, a particular problem is the relationship between warfarin‐vascular calcifications‐renal function decline. The mechanism entails vitamin K inhibition that indirectly inhibits matrix G1a protein, thus promoting vascular calcification and calciphylaxis. Progression of renal vascular calcifications is associated with renal function decline and higher hemorrhagic and thromboembolic risk.^9^, ^16^

**Table 3:** Dose adjustment for DOACs according to chronic kidney disease severity in patients with atrial fibrillation/venous thromboembolism (adapted from Potpara ‐ 9, Harel ‐ 22, Bhatia ‐ 13, Ghadban ‐ 23)

Recommended oral anticoagulant	CrCl (mL/min) estimated using the Cockroft‐Gault equation				
	≥50	30–49	15–29	<15	End‐stage renal disease on dialysis
	DOACs	DOACs	Warfarin/DOACs	Warfarin/DOACs (with caution)	
Warfarin	Preferable to adjust the dose function of time in therapeutic range, optimal ≥70%				
Dabigatran	150 mg twice daily 110 mg twice daily ≥80 years, or associated with P‐glycoprotein inhibitors, or high risk of hemorrhage	Idem	The United States (based only on FDA approval) ‐ 75 mg twice daily Europe ‐ NO	No	
Rivaroxaban	20 mg once daily	15 mg once daily (dose used by landmark trials recommended by small pharmacokinetic studies)		No	
Apixaban	5 mg twice daily 2.5 mg twice daily if any ≥2 of the following: age ≥ 80 years, body weight ≤ 60 kg and creatinine ≥1.5 mg/dL	Idem	2.5 mg twice daily	The United States – 2.5 mg twice daily Europe ‐ NO	The United States (FDA) ‐ 5 mg twice daily Europe ‐ NO
Edoxaban	60 mg once daily 30 mg once daily when ≥2 of the following criteria are met: body weight ≤ 60 kg, CrCl 30‐50 mL/min and therapy with Verapamil, Dronedarone or Quinidine is associated FDA black box warning for CrCl >95 mL/min	30 mg once daily		No	

Abbreviations: DOAC, direct oral anticoagulant; FDA, Food and Drug Administration.

*Direct oral anticoagulants* (DOACs) are a therapeutic option with evident advantages. However, in case of renal dysfunction, their use makes dose adjustment mandatory, as there is a variable degree of renal clearance (Table 3).^13^, ^14^ Dosage recommendations are derived from the analysis of data in the subgroups with AF and renal dysfunction from landmark trials (dabigatran—RE‐LY, rivaroxaban—ROCKET‐AF, apixaban—ARISTOTLE, edoxaban—ENGAGE‐AF TIMI 48). It is very important to mention that patients with creatinine clearance (CrCl) < 30 mL/min (<25 mL/min for apixaban) were excluded from these trials. Consequently, the guidelines adopted the indications for mild‐to‐moderate CKD, and recommended dose adaptation based on phase 3 trials.^18^, ^19^, ^20^, ^21^ Harel's meta‐analysis of the data from landmark trials in AF and VTE supported the use of DOACs vs warfarin in mild and moderate CKD due to their efficacy and safety uninfluenced by renal function.^22^ In the absence of clear data for severe CKD and ESRD dose adjustment can be based on manufacturer recommendations,^18^, ^19^, ^20^, ^21^ on small pharmacokinetics studies or observational studies (Table 3).^9^, ^23^. Thus, based on pharmacokinetic data, a dose of 75‐mg twice daily (BID) of dabigatran was approved by Food and Drug Administration (FDA) for patients with CrCl of 15 to 29 mL/min. Also, there are signals from small pharmacokinetic studies that recommend an additional reduction of rivaroxaban doses (10‐mg once daily (QD)) and edoxaban (25‐mg QD) in severe CKD.^9^ Also, the FDA issued a black box warning against the use of edoxaban in patients with CrCl >95 mL/min, considering the numerical but not statistically significant excess of ischemic strokes.^9^ Rivaroxaban is also not recommended in patients with VTE and CrCl <30 mL/min, and for CrCL 30 to 49 mL/min the recommended dose is 15‐mg BID for 21 days followed by 20‐mg QD.^22^ For patients on hemodialysis, but not for stage 5 nondialysis patients, the FDA allows apixaban 5‐mg BID, although according to pharmacokinetic data and label recommendations the dose of 2.5‐mg BID would ensure adequate plasma concentration.^9^, ^23^, ^24^ Labeling supports that rivaroxaban may be administered at a dose of 15 mg QD.^24^ There is no conclusive data for edoxaban.

One particular adverse effect is DOACs‐induced nephropathy, experimentally demonstrated and described by isolated reports; it is produced by the tubular obstruction caused by hematic cylinders, as well as by the activation of protease‐activated receptor 1.^25^ Finally, one important practical issue is the prospect for the renal dysfunction to worsen under anticoagulation treatment, defined as a reduction in CrCl ≥ 20%. Therefore, the analysis of data from landmark trials on DOACs supports the necessity of monitoring the renal function on a regular basis.^9^, ^20^

In clinical practice, compliance with all these recommendations is variable. A survey on the management of AF in CKD conducted in 41 European centers revealed that although renal function was monitored in >90% of the centers, patients were monitored at 1‐year intervals in 31.7% of the centers. Guideline recommendations for preferred OACs according to the severity of CKD and AF management in mild to moderate stages were followed. Alternatively, in ESRD, 31% of the centers did not use OACs and preferred AF rate control.^26^ Another study conducted in the United States found that in spite of guidelines and FDA‐issued recommendations, 60% of the patients with mild‐to‐moderate CKD receive lower doses of DOACs, which could account for the excess thromboembolic events.^27^

#### 3.1.2 Heparin treatment

Heparin, no matter of type, is administered according to the classic rules, depending on the associated disorder (acute coronary syndrome/VTE). Dose adjustment is necessary in advanced CKD and is primarily based on guideline recommendations (Table 4).^28^, ^29^, ^30^

**Table 4:** Dose adjustment for heparins according to chronic kidney disease stage (adapted from Hughes et al^15^, European Society of Cardiology guidelines^28^, ^29^, ^30^)

Anticoagulant	Dose adjustment function of eGFR (mL/min)		
	59‐30	29‐15	<15
Unfractionated heparin	Not necessary	Not necessary	Dose reduction by 33%: loading dose 60 IU/kg, maintenance 12 IU/kg/h, subsequent aPTT‐adjusted dosing
Enoxaparin	Not necessary (1 mg/kg/12 hours) VTE 1.5 mg/kg once daily (The United States)	1 mg/kg once daily anti‐Xa adjusted dosing (Anti‐Xa:Iia ratio 3.9)	
Dalteparin	ACS (120 IU/kg/12 hours) VTE (100 IU/kg/12 hours or 200 IU/kg once daily 1 month, then 150 IU/kg once daily 5 months, then oral anticoagulants/LMWH)	‐	
Tinzaparin	VTE (175 IU/kg once daily)	anti‐Xa adjusted dosing to eGFR <20 (Anti‐Xa:Iia ratio 2.8)	
Fondaparinux	VTE: 50% dose compared to the recommended dose per body weight ACS 2.5 mg once daily	VTE: not recommended for eGFR <30 ACS: not recommended for eGFR <20	
Argatroban	No dose adjustment is necessary (0.5‐2 μg/kg/min) ‐ renal clearance 15%		

Abbreviations: ACS, acute coronary syndrome; eGFR, glomerular filtration rate; VTE, venous thromboembolism.

*Unfractionated heparin (UFH)* is preferred because it has a short half‐life that allows for the anticoagulant effect to wear off within 1 to 4 hours, even in patients with severe renal dysfunction at high hemorrhagic risk. In addition to this, there is an antidote (protamine) used to rapidly reverse the effects of UFH, although the guidelines recommend UFH in severe CKD without adjustment of impaired renal clearance and interpatient variability of accumulation. Therefore, nephrology practice recommends decreasing the initial standard dose by 33%, and subsequently dose adjustment based on aPTT.^16^ In the NSTEMI guideline, indications are primarily based on dose adjustments.^29^ The primary argument is the high hemorrhagic risk per se in severe CKD.

*Low‐molecular‐weight heparin*s *(LMWHs)* are preferred owing to their pharmacokinetic predictability, ease of administration without the need for monitoring. Renal clearance is indirectly proportional to molecular weight, therefore it requires dose adjustments in CKD stages 4 and 5 (Table 4). For dosage adjustment purposes, it is recommended to monitor the activity of antifactor Xa (anti‐Xa level) in order to avoid underdosage and achieve optimal therapeutic level, respectively.^16^ Dosing indications are the result of either small‐scale open‐label studies, or analysis of CKD subgroups in the randomized trials, adopted by guidelines. Enoxaparin is the most commonly used low‐molecular‐weight heparin (LMWH) and the 1‐mg/kg QD regimen recommended in severe CKD the most studied. There is no data for dalteparin and tinzaparin in severe CKD; therefore, it is preferable to avoid administering them.^16^ Although preferred in cases of heparin‐induced thrombocytopenia, fondaparinux is not recommended in severe CKD.

### 3.2 Practical recommendations for optimizing the anticoagulant treatment

#### 3.2.1 OAC treatment

Balancing risks and benefits requires an optimal control of thromboembolic and/or hemorrhagic risk factors. Consequently, it is necessary to use the classic risk scores CHA_2_DS_2_‐VASc and HAS‐BLED. Attempts to introduce renal dysfunction in the CHA_2_DS_2_‐VASc (R_2_CHADS_2_, ATRIA) or HAS‐BLED scores were not associated with improved predictive value, in ESRD included.^8^, ^9^, ^20^ On the other hand, CKD certainly influences the quality of anticoagulation and risk of hemorrhage. To this end, Apostolakis et al have proposed the SAMe‐TT2R_2_ score and recommended warfarin for scores between 0 and 2 (if TTR > 65%‐70%) and DOACs from the start for scores ≥2.^31^ As for the HAS‐BLED score, it is deemed that 53% of CKD patients are at high risk of hemorrhage at values >2.^9^

*Anticoagulation monitoring* mandatorily requires optimal methods and an adequate monitoring calendar. The use of INR is the common method for *VKAs*, but its values are frequently unstable, rendering anticoagulation control quite challenging. The “start‐low go‐slow” rule for dosing VKAs and a 10%‐20% lower dose according to eGFR level is the logical and necessary approach. Weekly monitoring is yet another recommendation, but it proves difficult to implement in practice.^13^ However, frequent monitoring does allow to optimize the time in which INR values are within therapeutic range (TTR).^9^ Although TTR ≥ 70% is an independent predictor of lower risk of thromboembolism, major hemorrhage and mortality, in CKD its values are frequently suboptimal.

For *DOACs*, main issue is how to define kidney dysfunction for a correct dose adjustment. Landmark trials use CrCl calculated via the Cockroft‐Gault equation, which can overestimate renal function, particularly in advanced CKD and in patients weighing >100 kg.^16^ European Heart Rhythm Association (EHRA) believes that using the Chronic Kidney Disease Epidemiology Collaboration (CKD‐EPI) equation is safer and concurrently the method recommended by nephrologists.^20^ Note that the recommendations for use of the Cockroft‐Gault formula would be maintained for drugs with a relatively low safety factor.^16^ This aspect could create confusions in clinical practice, as it entails a reinterpretation of trial‐based evidence. However, reanalyzing the data from RE‐LY and ARISTOTLE trials did not reveal diminished safety and efficacy of DOACs. Clinical reality brings into discussion another essential issue, namely the low level of knowledge and application of the recommendations by practitioners. A recent real‐world study in the United States showed that in 43% of the patients the medication was overdosed, being associated with a 2.19 times increase in hemorrhagic risk, while in 13.3% of the patients the medication (apixaban) was underdosed, being associated with a 4.87 times increase in the risk of stroke.^32^ A prudent approach is renal function to be checked upon initiation of treatment with DOACs, after 3 months, and then every year, except for the high‐risk patients (elderly >75 years, women, patients with low body mass, frail or on dabigatran) who require monitoring at least every 6 months.^14^ The current position of EHRA is for the individualized estimation of recheck intervals using a simple calculation—if CrCl ≤ 60 mL/min: recheck interval in months is CrCl:10.^21^ Any condition worsening the renal function (infections, acute heart failure, potentially nephrotoxic medication, etc.) requires additional assessment. Regarding the optimal monitoring method, there are precise but less commonly used tests (for dabigatran—dilute thrombin time, for xabans—ecarin chromogenic assay).^20^

*Reversal of the antithrombotic effect of OACs* is achieved using fresh frozen plasma, prothrombin complex concentrate, recombinant factor VIIa, factor VIII inhibitor bypassing activity or a specific antidote. Factor VIII inhibitor bypassing activity is reported for use only in case of DOACs‐induced hemorrhage. Also, it is the only useful factor for reversal of dabigatran effects. Hemodialysis is reported as being useful only for dabigatran in small single‐center studies.^13^ If warfarin is used, INR ≥9 and there are no major bleeding events a single oral dose of vitamin K (2.5‐5 mg) is needed.^13^ In case of major bleeding, vitamin K 10 mg is administered parenterally every 12 hours, along with prothrombin concentrate or recombinant factor VIIa with rapid effect and without fluid overload.^13^ For dabigatran, in case of life threatening bleeding, the specific antidote Idarucizumab is indicated, with rapid effects after a single dose of 5 g i.v. The manufacturer does not recommend lowering the doses in CKD, and their efficacy in ESRD has not been tested.^13^ The FDA has recently approved Andexanet alfa as a reversal agent for rivaroxaban and apixaban. It is not approved for use in Europe. Ciraparantag (PER977)—a synthetic molecule with parenteral administration used as an antidote for all DOACs, UFH and enoxaparin is currently in phase 2 clinical trial and the results are expected soon.

#### 3.2.2 Monitoring the treatment with heparins

Activated Partial Thromboplastin Time (aPTT)‐based dose adjustment is still recommended for UFH to achieve an optimal therapeutic level (aPTT range 1.5‐2.0).^16^ For LMWH anti‐Xa level monitoring calibrated specifically for each therapeutic agent is compulsory in CKD stages 4 and 5. The initial monitoring is performed 2 to 4 hours before and postdose. Regular twice a week monitoring could be useful.^16^ Underdosage is defined as peak anti‐Xa levels <0.5 IU/mL. Optimal therapeutic levels are 0.1 to 0.3 IU/mL for prophylactic dosing and 0.4 to 1 IU/mL for therapeutic dosing.^16^ In the absence of clear guidelines and clinical trial‐based indications, adapted local protocols could be useful. Heparin‐induced thrombocytopenia is difficult to control in ESRD since fondaparinux is contraindicated. Another essential issue is the need to split the total LMWH dose into two equal doses in patients at high hemorrhage risk. This recommendation is derived from the Cochrane databases, showing that single‐dose regimens are not more effective, rather more convenient for the patient.^33^

## 4 OACS—BETWEEN EFFICIENCY AND SAFETY

### 4.1 What we know. Lessons from clinical trials

The majority of data refers to the relation between AF and CKD. As shown by observational studies and meta‐analyses, the prevalence of AF increases with the decline in renal function. The risk remains elevated in dialysis‐dependent CKD patients (×1.32‐1.46), corresponding to a prevalence of 6.42 to 9.91/1000 patient years.^34^ In predialysis patients, the rates range between 4% and 21%.^20^ Renal dysfunction is even more frequent in acute coronary syndromes, being present in 30% of STEMI and 40% of NSTEMI patients.^20^ For VTE, the risk is estimated to increase from 29% in mild CKD up to 134% in patients on dialysis.^8^ Overall, CKD is an independent risk factor for ischemic and hemorrhagic stroke, estimated at around 5%‐6%/year.^34^ Compared to the general population, the thromboembolic risk is 2.5 to 5.5 times higher depending on renal function status, while the hemorrhagic risk is at least double.^4^, ^9^, ^34^ The practical consequence is that although prophylactic anticoagulation is necessary, the benefits and safety may be affected compared to the general population.

Literature data on *VKAs* primarily refer to warfarin, which practically entails the extrapolation of data to other coumarin drugs. Most of the evidence comes from the analysis of AF patient subgroups. There is only one randomized control study (Stroke Prevention in Atrial Fibrillation III study) that included patients with stage 3 of CKD (42%) and analyzes warfarin compared to the population with normal renal function.^9^ Data analysis in CKD subgroup show that well‐adjusted doses reduce the risk of ischemic stroke and systemic embolism by 76% and 67%, respectively, without statistically significant differences in major bleeding rates. Other data on warfarin derive from registries and observational studies that include CKD subgroups. Overall, the results are consistent in terms of effectiveness in reducing the thromboembolic risk, the risk of cardiovascular/all‐cause mortality, as well as the risk of a fatal stroke.^34^ Meta‐analyses also favor the efficient and safe use of warfarin in nondialysis‐dependent CKD. In 2016, Dahal et al published a landmark meta‐analysis and stated that the use of warfarin in nondialysis‐dependent CKD reduces the risk of ischemic stroke and systemic embolism by 30%, all‐cause mortality by 35%, concurrently with an insignificant 15% increase in major hemorrhages compared to the group not receiving warfarin.^35^ As for ESRD without/on dialysis, there are no data from randomized trials, and the results regarding efficiency are inconsistent.^15^ Part of the studies report risks twice as high, particularly in the elderly. Some studies claim a neutral effect on ischemic stroke, while others report a reduction in the risk of stroke and all‐cause mortality in dialysis‐dependent CKD and composite endpoint (mortality, readmission for acute myocardial infarction or stroke) in ESRD.^9^, ^34^ Dahal's meta‐analysis supports the neutral effect on the risk of stroke, systemic embolism, and all‐cause mortality in ESRD and the tendency towards an insignificant increase of thromboembolic risk, and the neutral effect on mortality in ESRD on dialysis.^35^ Consistent across studies is the assertion of an increased risk of hemorrhage in dialysis patients.^4^, ^9^, ^34^ This is questionable considering the particularities of this subgroup with low life‐expectancy, twice as high mortality rate, multiple comorbidities, unstable INR and at high hemorrhagic risk per se, low adherence to treatment, and overlapping effect of dialysis methods.^9^

*DOACs* serve as a viable alternative to VKAs. As mentioned before, the available evidence for daily practice results from the analysis of subgroups with mild‐to‐moderate CKD in landmark trials.^9^, ^15^ For severe CKD, clinical data are lacking and pharmacokinetic studies recommend dose reduction.^15^ In ESRD, although rivaroxaban and apixaban appear to be safe, prospective clinical data are needed.^15^ Also, the meta‐analysis by Nielsen et al shows that the efficacy and safety of DOACs are not influenced by CrCl up to 30 mL/min (25 mL/min for apixaban) and that they are comparable. Apixaban is also with lower bleeding rates in these patients compared with warfarin.^36^ Moreover, Ruff et al in another meta‐analysis further states that in CKD, DOACs reduce by 19% the thromboembolic risk, all‐cause mortality, and the risk of intracerebral hemorrhage, but increase the risk of gastrointestinal bleeding compared with warfarin.^37^ Finally, the extensive analysis conducted by Harel concluded that dabigatran, apixaban and rivaroxaban demonstrate similar efficacy and safety to warfarin.^22^

An important issue is maintaining an optimal risk‐benefit ratio in patients with stable CKD and episodes of acute renal failure. Studies regarding rivaroxaban and apixaban report consistent beneficial effects in these conditions.^4^ A prospective multicenter noninterventional real‐world European registry is currently ongoing (Factor XA‐Inhibition in REnal Patients with Non‐valvular Atrial Fibrillation‐XARENO). Its goal is to collect data from at least 2500 patients with nonvalvular AF and eGFR 15 to 49 mL/min/1.73 m^2^, who will receive rivaroxaban (1000 patients), warfarin or no OACs and will be monitored for at least 12 months in terms of medication efficiency and safety.^38^

### 4.2 What we do not know

*Heparin‐based anticoagulation* is challenging in patients undergoing dialysis. Poor anticoagulation can affect the quality of dialysis session. Patients undergoing dialysis frequently receive OACs or platelet antiaggregants. That is why systemic anticoagulation becomes a sensitive matter in long‐term hemodialysis. UFH is used according to a classic protocol, while LMWHs are administered as a bolus dose at the start of dialysis and heparin‐like molecules are administered in particular situations. The attitude towards LMWH use in hemodialysis patients is variable. The FDA has not approved their use in the United States, while other local guidelines either recommend them, or do not provide clear indications.^39^ According to several small‐scale studies and a meta‐analysis published in 2004, LMWHs appear to be safe and as effective as UFH. For these reasons, a protocol for the prospective monitoring and use of heparin‐based anticoagulation in hemodialysis patients with VTE was initiated in 2015 and is still ongoing.^39^

*Oral anticoagulation*. Although OACs are necessary in severe CKD and ESRD, evidence is inconsistent, often of unsatisfactory quality, and it draws attention to the potentiation of hemorrhagic risks. There are available data for warfarin, but data for DOACs are entirely inconclusive. In patients with AF, the existing guidelines have a different approach. The American guideline recommends warfarin even in dialysis patients with CHA_2_DS_2_‐VASc score ≥2 and INR between 2 and 3 (class IIa, LOE B) and does not allow the use of dabigatran and rivaroxaban in ESRD and dialysis patients. Equally important is the recommendation to initiate therapy after a strictly individualized evaluation of the risk‐benefit ratio.^19^ The ESC guideline recommends the preferential use of DOACs in patients with eGFR 5 to 29 mL/min/1.73 m^2^ and does not provide information for dialysis. For CKD requiring renal transplantation, there are no randomized trials and thus DOACs should be administered according to eGFR also considering the possible interactions with the immunosuppressant medication.^18^ Both guidelines highlight how difficult it is to provide standard recommendations in the absence of randomized controlled trials.^9^

Consequently, the initiation of anticoagulation therapy in severe CKD and ESRD is still a debate topic. Practitioners are confused by the information based on observational studies with irregular designs or inaccurate administrative registries, with extremely variable results.^9^ Systematic analyses and meta‐analyses can no longer provide accurate data, even when conducting sophisticated data analysis. Uncertainty is even higher in VTE, for which there are no guideline recommendations. Another problem is that of OACs for primary prevention of thromboembolic events in AF, particularly in stage 5 and dialysis patients.^9^, ^34^

Cardiologists tend to extrapolate guideline recommendations to patients without CKD, while nephrologists are reticent in this respect. The latest 2011 KDIGO (Kidney Disease: Improving Global Outcomes)guidelines state that only nephrologists should recommend OACs as primary prevention in ESRD and dialysis patients, based on a strictly individualized algorithm. A prudent approach to secondary prevention is to give OAC to all ESRD patients, without absolute contraindications.^40^ The use of DOACs is nonstandardized, possible in low apixaban/rivaroxaban doses in patients with ESRD at very high risk of ischemic stroke and with warfarin intolerance or suboptimal TTR, or in patients with recurrent stroke on adequate warfarin treatment.^40^

There are several ongoing trials, comparing DOACs and warfarin in AF and CKD stages 4 and 5, as well as randomized studies focusing on the safety of OACs in patients with AF and dialysis (Oral Anticoagulation in Haemodialysis Patients‐AVKDIAL, Compare Apixaban and Vitamin‐K Antagonists in Patients With Atrial Fibrillation and End‐Stage Kidney Disease‐AXADIA, Trial to Evaluate Anticoagulation Therapy in Hemodialysis Patients With Atrial Fibrillation‐RENAL‐AF).^9^ It is also possible for new DOACs to be developed, with particular pharmacokinetic features adapted to CKD stages. Although the percutaneous left atrial appendage closure in patients with contraindications for anticoagulation such as ESRD and dialysis supposedly is an alternative according to preliminary reports, the data require validation.^15^

## 5 CONCLUSIONS

There are therapeutic resources for CKD requiring anticoagulation, but evidence from randomized controlled trials is limited for mild and moderate stages. DOACs should be avoided in severe forms of CKD. Although VKAs have the downside of being unpredictable, the major advantage is that their use can be extended all the way through the terminal stage and can be easily reversed if necessary. When faced with such a complex patient, initiating and maintaining anticoagulation becomes a challenging task, which needs to be based on the *primum non nocere* principle.^34^ The strict determination of the individual profile, adequate selection of the type of anticoagulant and careful monitoring are some extremely useful indications for overcoming management challenges. Although there is a need for cardiorenal consensus, based on the current data the fact remains that DOACs are preferred in stages 1 to 3. In stage 4, the choice between DOACs vs warfarin will consider the pharmacokinetics of the drugs and patient characteristics. Warfarin remains the first‐line treatment in ESRD, although in this case the actual decision to use or not to use anticoagulation is strictly individualized. Anticoagulation with heparin is safe in nondialysis‐dependent CKD if optimal monitoring is ensured, but remains a challenge in the hemodialysis patients.

## CONFLICT OF INTEREST

The authors declare no potential conflict of interests.
